# Comparison of stranded and non-stranded RNA-seq transcriptome profiling and investigation of gene overlap

**DOI:** 10.1186/s12864-015-1876-7

**Published:** 2015-09-03

**Authors:** Shanrong Zhao, Ying Zhang, William Gordon, Jie Quan, Hualin Xi, Sarah Du, David von Schack, Baohong Zhang

**Affiliations:** Clinical Genetics and Bioinformatics, Pfizer Worldwide Research & Development, Cambridge, MA 02139 USA; Precision Medicine – Bioanalytical, PTx Clinical R&D, Pfizer Worldwide Research & Development, Cambridge, MA 02139 USA; Computational Sciences Centers of Excellence, Pfizer Worldwide Research & Development, Cambridge, MA 02139 USA

**Keywords:** RNA-seq, Gene quantification, Stranded, Non-stranded, Transcriptomics, Transcriptome profiling, Gene overlap

## Abstract

**Background:**

While RNA-sequencing (RNA-seq) is becoming a powerful technology in transcriptome profiling, one significant shortcoming of the first-generation RNA-seq protocol is that it does not retain the strand specificity of origin for each transcript. Without strand information it is difficult and sometimes impossible to accurately quantify gene expression levels for genes with overlapping genomic loci that are transcribed from opposite strands. It has recently become possible to retain the strand information by modifying the RNA-seq protocol, known as strand-specific or stranded RNA-seq. Here, we evaluated the advantages of stranded RNA-seq in transcriptome profiling of whole blood RNA samples compared with non-stranded RNA-seq, and investigated the influence of gene overlaps on gene expression profiling results based on practical RNA-seq datasets and also from a theoretical perspective.

**Results:**

Our results demonstrated a substantial impact of stranded RNA-seq on transcriptome profiling and gene expression measurements. As many as 1751 genes in Gencode Release 19 were identified to be differentially expressed when comparing stranded and non-stranded RNA-seq whole blood samples. Antisense and pseudogenes were significantly enriched in differential expression analyses. Because stranded RNA-seq retains strand information of a read, we can resolve read ambiguity in overlapping genes transcribed from opposite strands, which provides a more accurate quantification of gene expression levels compared with traditional non-stranded RNA-seq. In the human genome, it is not uncommon to find genomic loci where both strands encode distinct genes. Among the over 57,800 annotated genes in Gencode release 19, there are an estimated 19 % (about 11,000) of overlapping genes transcribed from the opposite strands. Based on our whole blood mRNA-seq datasets, the fraction of overlapping nucleotide bases on the same and opposite strands were estimated at 2.94 % and 3.1 %, respectively. The corresponding theoretical estimations are 3 % and 3.6 %, well in agreement with our own findings.

**Conclusions:**

Stranded RNA-seq provides a more accurate estimate of transcript expression compared with non-stranded RNA-seq, and is therefore the recommended RNA-seq approach for future mRNA-seq studies.

**Electronic supplementary material:**

The online version of this article (doi:10.1186/s12864-015-1876-7) contains supplementary material, which is available to authorized users.

## Background

RNA-sequencing (RNA-seq) is a next-generation sequencing technique that allows an in-depth look into the transcriptome [1–3]. Compared with microarray-based profiling, RNA-seq can detect the expression of low abundance transcripts and subtle changes under different conditions. RNA-seq has a wider dynamic range and avoids some of the technical limitations in a microarray experiment such as varying probe performance, cross-hybridization, limited dynamic range of individual probes, and nonspecific hybridization [4, 5]. RNA-seq is not limited to known transcripts and thus delivers unbiased and unprecedented information about the transcriptome and gene expression levels. With decreasing sequencing cost, RNA-seq is becoming an attractive approach to profile gene expression levels or specific transcript abundance, and to analyze differential gene expression between biological conditions.

While RNA-seq is emerging as a powerful technology in transcriptome profiling, one significant shortcoming of the standard RNA-seq protocol is that it loses the strand of origin information for each transcript. Synthesis of randomly primed double-stranded cDNA followed by the addition of adaptors for next-generation sequencing leads to the loss of information on which strand the original mRNA template is coming from, and without that information it becomes difficult to accurately determine gene expression from overlapping genes [6], i.e., those genes that have at least partially overlapping genomic coordinates, but are transcribed from opposite strands. Knowing the strand information of the cDNA is essential to determine from which of the overlapping genes the RNA transcript originates.

**Fig. 1:**
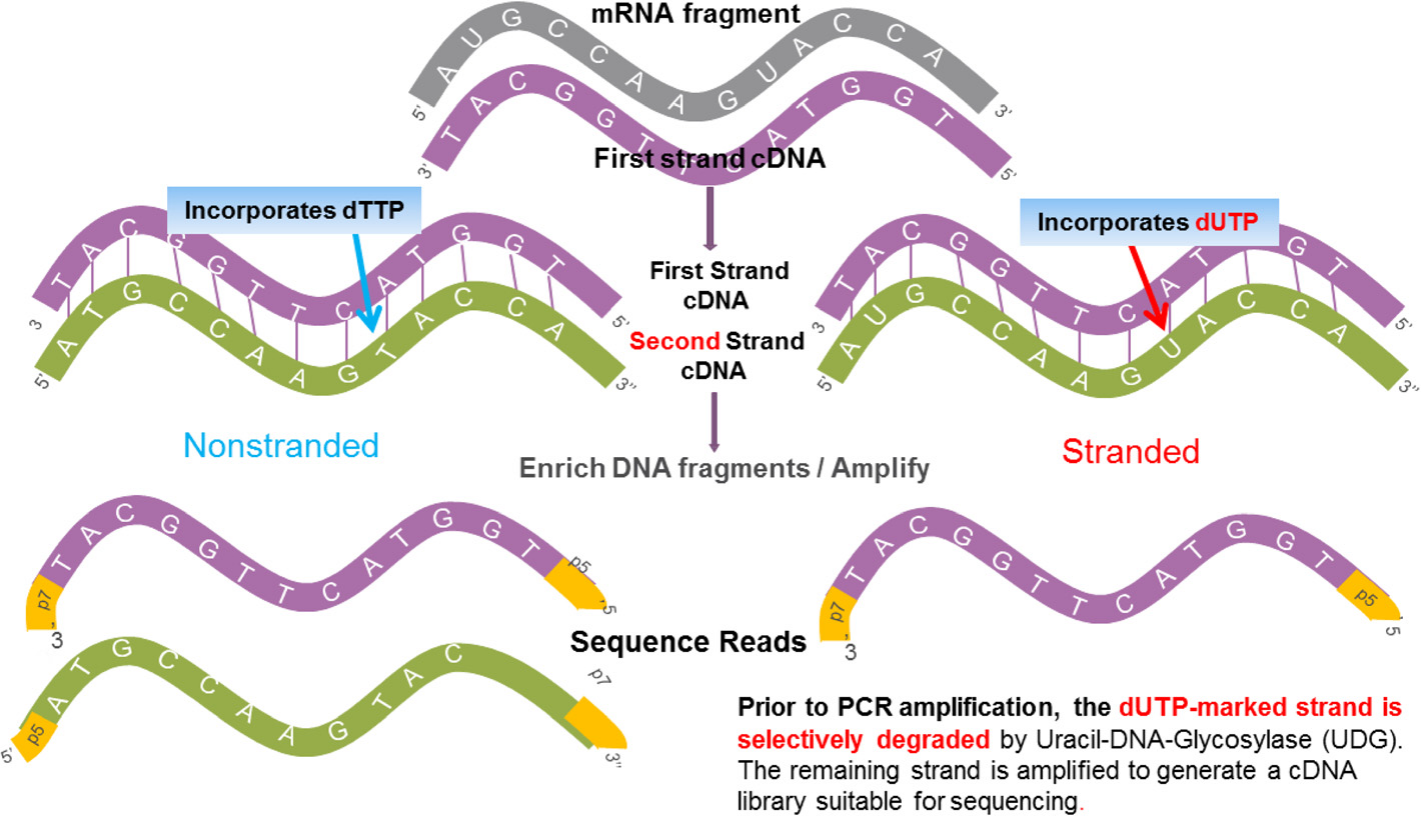
Non-stranded versus stranded RNA-seq protocol. The stranded protocol differs from the non-stranded protocol in two ways. First, during cDNA synthesis, the second-strand synthesis continues as normal except the nucleotide mix includes dUTPs instead of dTTPs. Second, after library preparation, a second-strand digestion step is added. This step ensures that only the first strand survives the subsequent PCR amplification step and hence the strand information of the libraries

It is now possible to retain the information pertaining to strand origin by modifying the standard RNA-seq protocol; this is known as strand specific RNA-seq, or stranded RNA-seq. Recently, multiple protocols for stranded RNA-seq have been published [7–10]. Seven protocols were comprehensively evaluated by researchers at the Broad Institute [10] and the authors found marked differences in strand specificity, library complexity, evenness and continuity of coverage, agreement with known annotations and accuracy for expression profiling. Weighing each method’s performance and ease of use, the authors identified dUTP second-strand marking [7] as one of the leading protocols (Fig. 1). The dUTP second-strand marking method, or dUTP method for short [7], uses dUTPs instead of dTTPs during the synthesis of the second strand in the cDNA synthesis step of sequencing library preparation. Prior to polymerase chain reaction (PCR) amplification, the second strand, harboring uracils, is degraded using uracil-N-glycosylase. With the second strand degraded, only the first strand is amplified in the subsequent PCR. This protocol was evaluated as superior in terms of both its simplicity and the data quality [10]. According to the protocol in Fig. 1, because the sequence reads generated from the dUTP method are reverse complementary to the originating mRNA transcripts, the strand information is retained throughout the sequencing process.

This new methodology is now emerging as a powerful tool for transcript discovery, genome annotation, and expression profiling [11, 12]. Previous reports demonstrated that data from stranded libraries are more reliable than data from non-stranded libraries and can correctly evaluate the expression of both antisense RNA and other overlapping genes [11]. Maintaining strand orientation also allows identification of antisense expression, an important mediator of gene regulation. The ability to capture the relative abundance of both sense and antisense expression provides insight into regulatory interactions that might otherwise be missed [12]. With the ability to unlock new information on global gene expression, stranded RNA-seq holds the key to a deeper understanding of the transcriptome.

To allow for efficient transcript/gene detection, highly abundant ribosomal RNAs (rRNAs) must be removed from total RNA before sequencing [13]. One standard solution is to enrich for the polyadenylated (polyA) tail attached RNA transcripts (so-called mRNA-Seq) with oligo (dT) primers. Another approach removes rRNA through hybridization capture of rRNA followed by binding to magnetic beads for subtraction. For most transcriptome studies, mRNA-seq is commonly used, as the sequencing depth required is lower when focusing only on the protein coding fraction of the transcriptome. In this paper, we performed a side-by-side comparison of stranded and non-stranded mRNA-seq by sequencing the same samples using both protocols. We investigated and characterized gene overlap in our RNA-seq dataset, as well as performed theoretical analysis of the number of overlapping genes based on genome annotation in Gencode Release 19 [14]. We demonstrate that stranded RNA-seq improves the accuracy of gene quantification, and this is especially critical for accurate gene expression quantification of antisense genes.

## Results and discussion

**Fig. 2:**
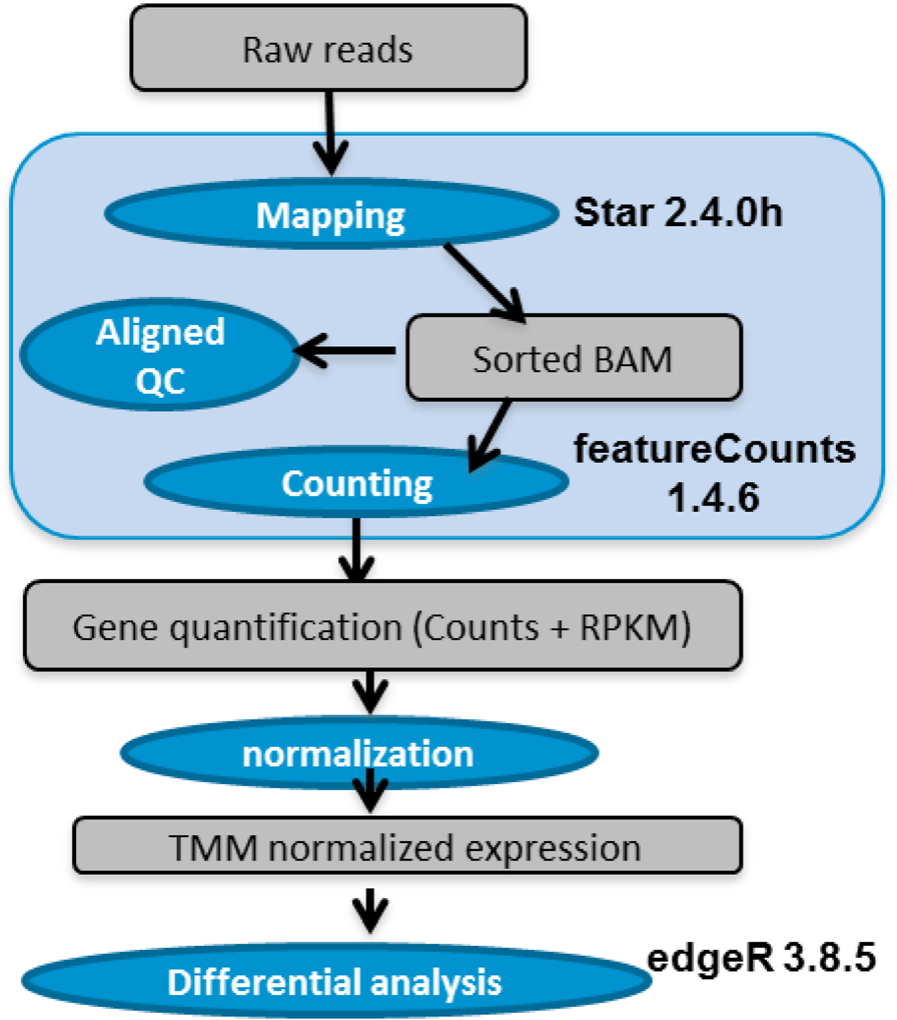
Workflow for RNA-seq data analysis

The sample preparation, sequencing, and data analysis are detailed in the Methods section. In brief, we collected blood from five healthy donors into Paxgene RNA tubes and pooled all samples. Four replicate samples (labeled as PFE1, PFE2, PFE3, and PFE4) were sequenced using both stranded (denoted as S) and non-stranded (denoted as NS) protocols. We note that these samples are considered technical replicates and therefore represent an ideal scenario with minimal variation. In this paper, we use the name convention “*Sample_Protocol*” to label each RNA-seq dataset. For instance, *PFE1_S* represents the sample PFE1 sequenced by stranded RNA-seq. For RNA-seq data analysis, we implemented an in-house pipeline in the Pfizer High Performance Computing environment as shown in Fig. 2. Raw sequence reads were mapped to human genome hg19 by STAR [15], and the uniquely mapped reads were counted by featureCounts [16] in the Subread package. Multiple mapped reads were excluded from counting, and then differential analysis was performed by the R packages edgeR [17] and Limma/voom [18]. Gene quantification and differential analysis results are dependent upon the initial choice of gene annotation [19–21]. In the previous paper [21], we evaluated the impact of different annotations on RNA-seq data analysis, including RefGene [22], UCSC [23], and Ensembl [24]. Gencode annotation [14] is based upon Ensembl but with improved coverage and accuracy, and it is used by the ENCODE consortium as well as many other projects (e.g., 1000 Genomes) as the reference gene set. In this evaluation, we therefore also chose the Gencode annotation, and the conclusions in this paper should largely (or for the most part) hold true when other gene annotations are used (data not shown).

### Read mapping and counting

**Fig. 3:**
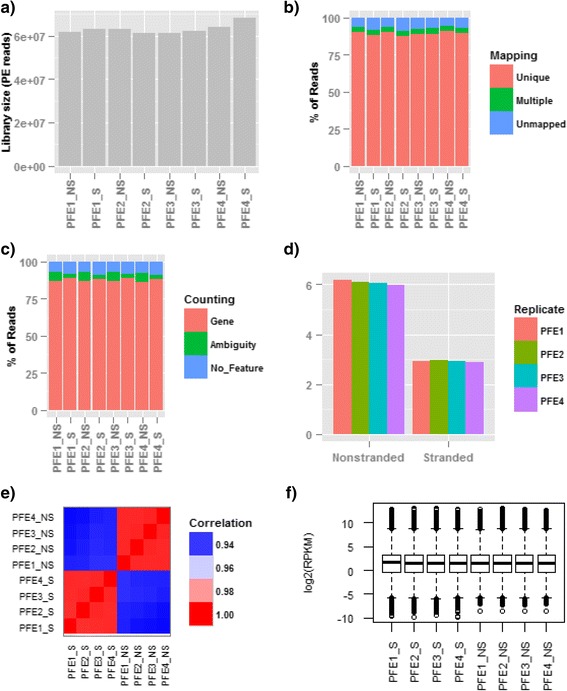
Metrics for RNA-seq. **a**) The sequencing library size; **b**) the mapping summaries for sequence reads; **c**) the counting summaries for uniquely mapped reads; **d**) the ambiguous reads arising from gene overlapping; on average, the percentage of ambiguous reads drops approximately 3.1 % from non-stranded to stranded RNA-seq, and this drop roughly represents the overlapping arising from opposite strands; **e**) the correlation for gene expression profile among those eight samples; the samples are clearly clustered by sequencing protocol; **f**) the boxplot of gene expression

Each replicate sample was sequenced by both non-stranded and stranded RNA-seq. The summaries for sequencing depth, mapping, and counting are shown in Fig. 3 and listed in Additional file 1: Table S1. For each sequenced library, there are over 60M paired-end reads (Fig. 3a) available for alignment and gene quantification. Overall, about 87–91 % of reads uniquely map to genomic regions, while approximately 3.5 % of reads map equally well to multiple locations. A remainder of ~5–8 % of reads fails to map to any locus in the human genome (Fig. 3b). In principle, non-stranded and stranded RNA-seq should have comparable mapping statistics for the same sample. However, as shown in Fig. 3b, the percentage of uniquely mapped reads in non-stranded RNA-seq is slightly higher than in stranded RNA-seq. After further investigation, we found that the average fragment size in non-stranded libraries is ~30 bp longer than in stranded sequencing. This may be caused by special treatment and the PCR enzyme in Illumina’s kit. As a result, in stranded sequencing, there are an estimated 4 % of fragments whose sizes are even shorter than the sequence read length used in this study (i.e., 100 bp). Therefore, sequence reads derived from short fragments end up contaminated with nucleotide bases from adapters and thus might fail to map to the genome because of too many mismatches.

As shown in Fig. 3c, the majority of uniquely mapped reads are counted towards genes in both stranded and non-stranded RNA-seq as expected for mRNA-seq. About 7–8 % of mapped reads do not match to any gene and thus are excluded from gene quantification. The ambiguous reads in Fig. 3c are those reads mapped to overlapping gene regions, either on the same strand or from the opposite strands. To highlight the genomic loci with genes overlapping on the two opposite strands, the read ambiguity in Fig. 3c is zoomed out and shown in Fig. 3d. The read ambiguity in stranded RNA-seq arises only from overlapping genes transcribed from the same strand. In contrast, for non-stranded RNA-seq, the ambiguity arises from both the overlapping genes on the same strand and also from the opposite strands. For the four stranded RNA-seq samples, the read ambiguity is an average of 2.94 % (Fig. 3d and Additional file 1: Table S1), while for the four non-stranded RNA-seq samples it is 6.1 % (Fig. 3d and Additional file 1: Table S1). Compared with non-stranded RNA-seq, the percentage of ambiguous reads in stranded RNA-seq drops by approximately 3.1 %, and this drop roughly represents the magnitude of gene overlap from the two opposite strands. As we demonstrate below, the gene overlap from our RNA-seq dataset is also consistent with our theoretical estimation.

The correlation for gene expression levels among the eight samples studied is plotted in Fig. 3e. The samples are clearly clustered by sequencing protocol, and while the correlation for samples prepared with the same protocol is nearly 1, the correlation for samples sequenced by the two different protocols is around 0.93. The correlation plot in Fig. 3e indicates underlying gene expression profile differences between the stranded and non-stranded RNA-seq methods. The distribution of gene expression in each sample is shown in the boxplot in Fig. 3d (note the y-axis is log2(RPKM)). Overall, the distribution across samples is very similar. The 1^st^ quartile, median, and 3^rd^ quartile are approximately 0.77 RPKM, 3.0 RPKM, and 9.6 RPKM, respectively. The gene expression distribution plot in Fig. 3d is a good reference to evaluate whether gene expression is relatively low, medium, or high.

### Theoretical estimate of frequency and magnitude of gene overlap

**Fig. 4:**
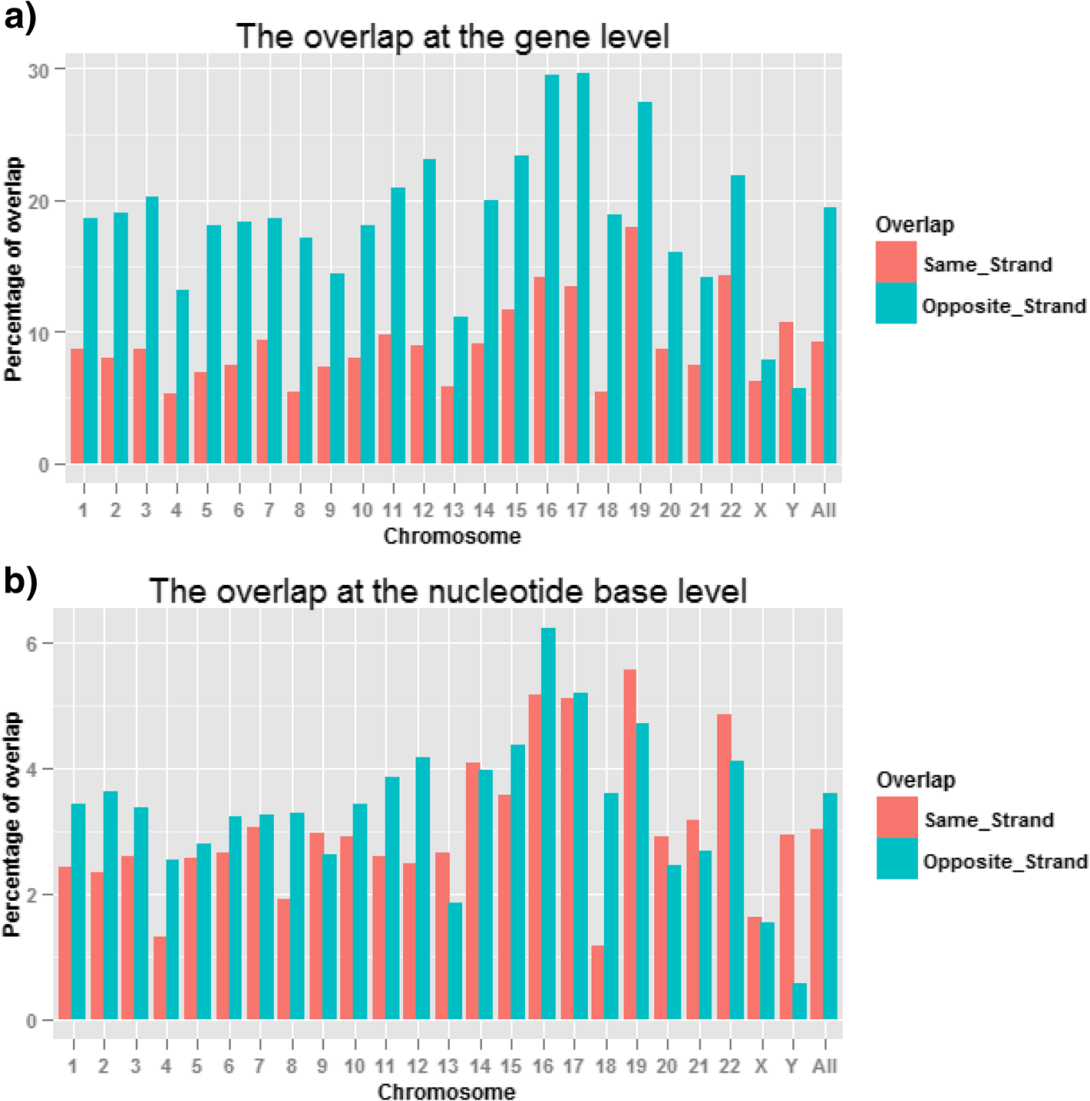
Estimated gene overlaps in Gencode Release 19. **a**) The same strand and opposite strand overlaps at the gene level; about 19 % of genes overlap with one or more genes at the opposite strand; **b**) the overlaps at the nucleotide base level. On average, the estimated overlapping at the same and opposite strands are 3 % and 3.6 %, respectively, and agree well with the practical RNA-seq dataset shown in Fig. 3d

Every gene in Gencode Release 19 has genomic coordinates, and the frequency of overlapping genes can thus be calculated (Fig. 4 and Additional file 1: Tables S2 and S3). There are more than 57,800 annotated genes in Gencode Release 19. Figure 4a shows the overlaps at the gene level. For all chromosomes, the frequency of opposite strand overlap is greater than the same strand overlap in terms of the number of overlapping genes. On average, approximately 9 % of genes overlap at the same strand, while for the overlap from opposite strands, the overlap increases to approximately 19 %. Stranded RNA-seq can resolve the read ambiguity in overlapping genes that are transcribed from opposite strands. Accordingly, 19 % of genes (i.e., 11,000 genes) in Gencode Release 19 are expected to have more accurate gene quantification in stranded RNA-seq than in non-stranded RNA-seq. As more and more novel genes are discovered in the genome, it is expected that additional genes will have overlapping genomic loci.

Genomic loci with longer overlapping genes will produce more transcript reads that cannot be uniquely assigned to either strand when using non-stranded RNA-seq. To further estimate the impact of overlap on gene quantification, we quantified the overlaps at the nucleotide level (Fig. 4b). On average, the estimated overlaps at the same and opposite strands are 3 % and 3.6 %, respectively, and this agrees very well with our practical RNA-seq data. According to our stranded RNA-seq dataset, the read ambiguity in overlapping genes at the same strand is 2.94 % (Fig. 3d and Additional file 1: Table S1), which is very close to the theoretical estimation (Fig. 4b and Additional file 1: Table S3). In Fig. 3d, the opposite strand overlap in our actual RNA-seq dataset is 3.1 %, slightly lower than the theoretical 3.6 % (Fig. 4b). It should be pointed out that the theoretical estimation is based upon the assumption that all genes in the Gencode annotation database are uniformly expressed. In an actual RNA sample, the expression level varies from gene to gene, including genes that are not expressed at all. In addition, with our chosen sequencing protocol, a transcript is not picked up if it does not have a polyA tail at the 3’ end. Still, the theoretical estimation in Fig. 4b explains very well the counting summary for ambiguous reads in Fig. 3d and Additional file 1: Table S1. In practice, the overlap in actual samples may be higher or lower than our theoretical estimation depending upon the gene expression profile in a sample.

**Fig. 5:**
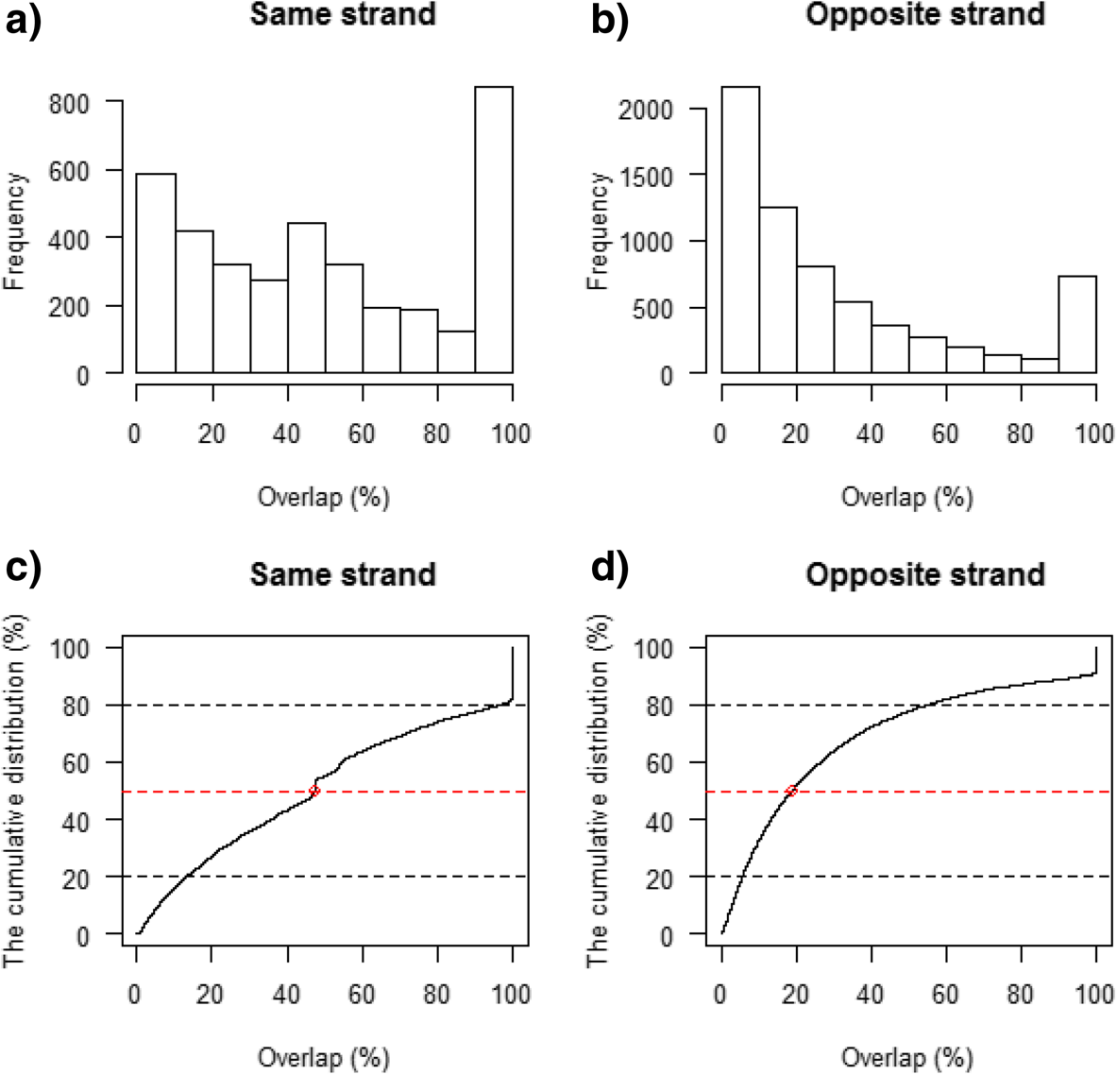
Histograms and cumulative distributions for all pairs of overlapping genes. The ratio (i.e., the overlapping percentage) for each pair of genes is calculated by dividing the length of overlapping exons by the exon length of the shorter gene of the pair

We also quantified the degree of gene overlap by analyzing all pairs of overlapping genes. First, we identified the common or overlapping exon regions between any two overlapping genes. Then, the shorter gene was selected and the ratio (i.e., the overlapping percentage) was calculated by dividing the length of overlapping exons by the exon length of the shorter gene. A total of 6582 overlapping gene pairs were identified from opposite strands and the number was 3718 at the same strand. The histograms and cumulative distributions of overlaps are shown in Fig. 5. The histograms (Fig. 5a and b) indicate the extent of overlap ranges from partial to complete. There are 582 genes that are 100 % contained within other genes at the same strand, while 654 genes are completely contained within the same genomic locus of another gene from the opposite strand. The cumulative distributions in Fig. 5c and 5d describe the probability of having an overlapping gene pair with an overlap less than or equal to a given threshold. For the same strand overlap, the medium overlap is approximately 47.4 %, while for the opposite strand overlap, its medium is approximately 18.7 %. In general, the magnitude of overlap for the same strand is greater than the overlap from the opposite strands.

### Differential analysis

**Fig. 6:**
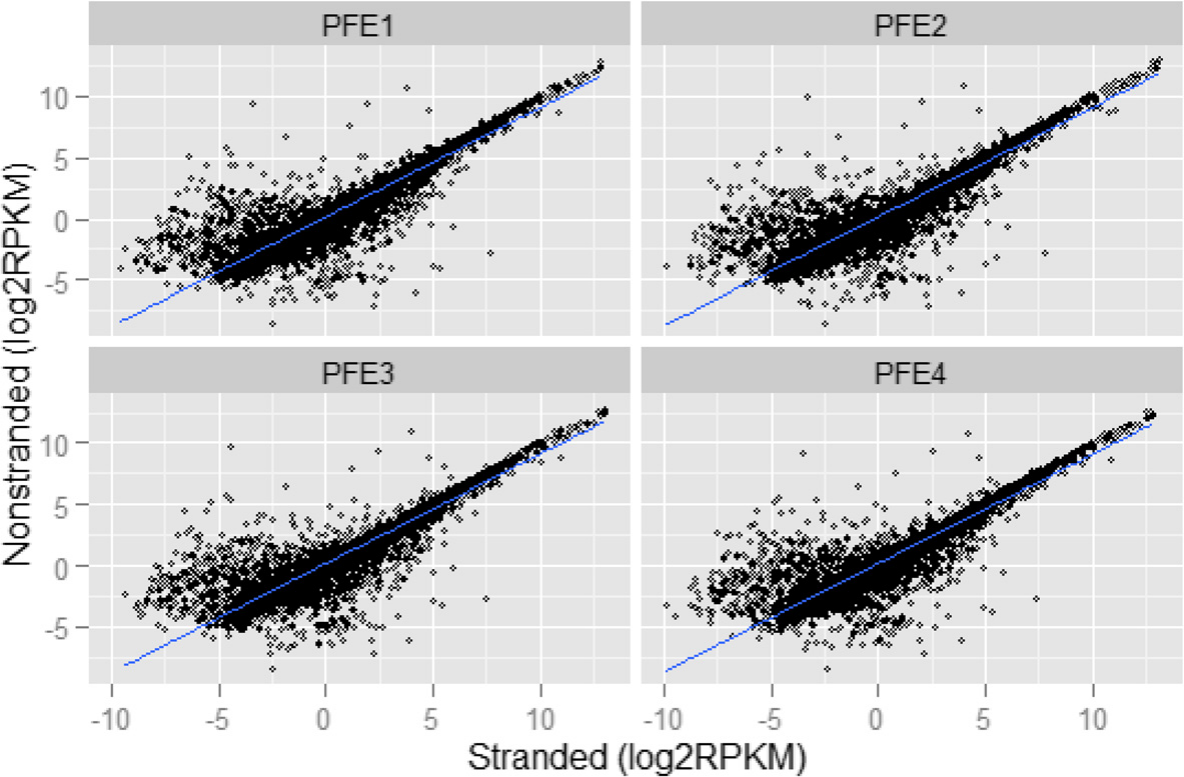
Scatter plots of gene expression profiles between stranded and non-stranded RNA-seq. For samples PFE1, PFE2, PFE3, and PFE4, the scattering patterns are consistent. While the majority of genes are arrayed along the diagonal lines, there are still many genes whose expression levels were dramatically impacted by sequencing protocols. The x- and y-axis represent Log2(RPKM)

The scatter plots of the gene expression profiles for the four replicate samples are shown in Fig. 6. For comparison, the all-against-all scatter plots for stranded and non-stranded samples are shown in Additional file 1: Figures S1 and S2, respectively. For technical replicates sequenced by the same protocol, all data points are arrayed clearly along the diagonal lines with a relatively large variation only for genes with low expression. However, when comparing the same samples sequenced by the two different protocols, there are many genes that are far away from the diagonal lines along the length of the axis in Fig. 6. For samples PFE1, PFE2, PFE3 and PFE4, the scatterplot patterns are very consistent as expected from technical replicates. As observed in Fig. 6, for a large number of genes, the sequencing protocol has a dramatic impact on the final gene quantification results. It is not unusual that there are genes whose expression levels are high in one protocol, but very low or even zero in the other protocol.

**Fig. 7:**
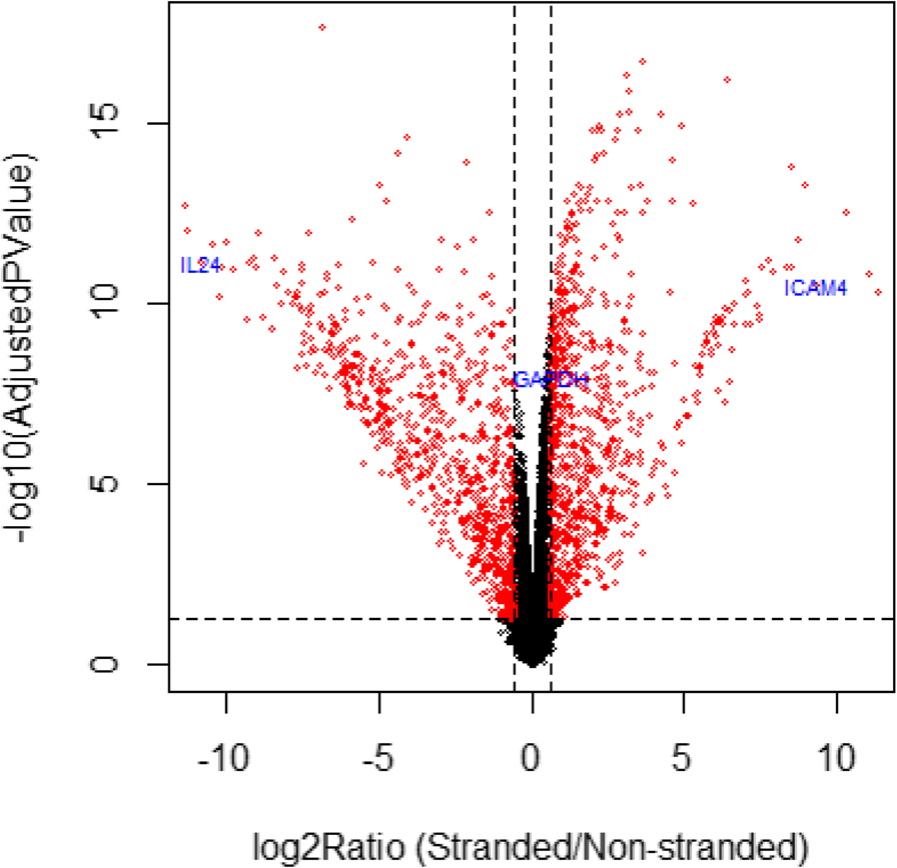
Differential analysis results for the comparison between stranded and non-stranded RNA-seq. Every point in the plot corresponds to a gene. The x-axis represents the log2 fold change of stranded over non-stranded, while the y-axis (-log10 (AdjustedPValue)) corresponds to the significance of a statistical test. All significant genes are colored in red. The criteria for significance are as follows: (1) an adjusted p value <0.05 (the horizontal dotted line); and (2) a fold change greater than 1.5 (the two vertical dotted lines)

To identify genes with large expression differences between stranded and non-stranded RNA-seq, we performed a differential expression analysis using R packages edgeR [17] and Limma/voom [18]. The raw read counts generated by featureCounts [16] were normalized by TMM (trimmed mean of M-values) in edgeR first, followed by standard differential analysis. The statistical test results are summarized in Fig. 7. Each point in the plot corresponds to a gene. The x-axis represents the log2 fold change of stranded versus non-stranded, while the y-axis (-log10(Adjusted PValue)) corresponds to the significance of statistical test. A total of 1751 significant genes were identified to be differentially expressed (DE) and are colored in red in Fig. 7. The criteria for significance are as follows: (1) an adjusted p value <0.05 (the horizontal dotted line in Fig. 7); and (2) a fold change greater than 1.5 (the two vertical dotted lines in Fig. 7). Of those significant genes, 941 genes (top right corner) have higher expression in stranded than in non-stranded sequencing, while 841 genes (top left corner) are down regulated, having lower expression in stranded than in non-stranded RNA-seq. The large number of differential expression genes in Fig. 7, together with the scatter plots in Fig. 6 and the correlation plot in Fig. 3e, clearly demonstrates the substantial impact of sequencing protocols on gene quantification.

**Fig. 8:**
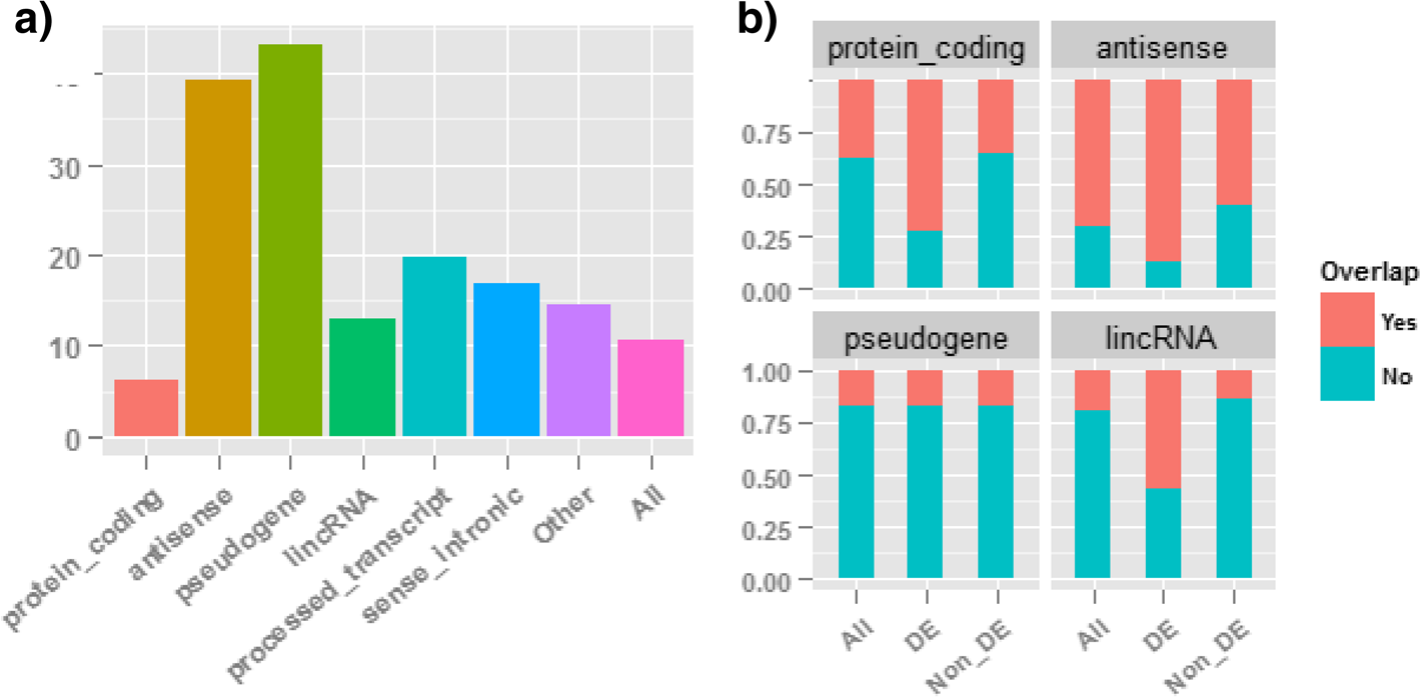
The association between differential expression and gene overlap is gene-type dependent. **a** The percentage of genes that are differentially expressed in each gene category. Antisense and pseudogene are enriched. The y-axis represents percentage. **b** The dependency between differential expression and gene overlap from opposite strands. For protein coding, antisense and lincRNA gene types, the overlap is significantly higher in DE genes than in Non_DE genes

A gene is considered to be expressed if its maximal expression across all eight samples is greater than 1 CPM (count per million), and accordingly, a total of 16,443 expressed genes survived this filtering. All genes that have appreciable expression and those 1751 DE genes in Fig. 7 can be further broken down into the gene categories shown in Table 1. The detailed description of each gene category from Gencode annotation was described previously [25]. As shown in Table 1 and Additional file 1: Figure S3, over 80 % of expressed genes are protein coding, while both antisense genes and pseudogenes account for roughly 5 % each. However, for DE genes, the percent of protein coding drops to 46 %, but both the antisense and pseudogene categories increase to ~20 % each. Thus, the differential expression we observe is associated with gene type. Globally, 10.65 % of genes are differentially expressed when comparing the stranded and the non-stranded RNA-seq data. However, for antisense genes and pseudogenes, the ratios jump to 39 % and 43 %, respectively (Table 1 and Fig. 8a). To test whether the apparent enrichment of antisense genes and pseudogenes is statistically significant, the built-in binomial proportions test *prop.test* in R was used. The calculated p values are smaller than 2.2E-16 for both gene categories, indicating the enrichment is not by chance.

**Table 1:** The association between differential expression and gene overlapping is gene-type dependent

Gene_type	Differential analysis					Overlapping			
	All genes		DE genes		Ratio^a^ (%)	DE genes		Non_DE genes	
	#	%	#	%		No	Yes	No	Yes
Protein_coding	13219	80.39	810	46.26	6.13	226	584	8082	4327
Antisense	924	5.62	363	20.73	39.29	48	315	225	336
Pseudogene	845	5.14	365	20.85	43.20	304	61	398	82
LincRNA	764	4.65	100	5.71	13.09	43	57	571	93
Processed_transcript	182	1.11	36	2.06	19.78	7	29	76	70
Sense_intronic	113	0.69	19	1.09	16.81	12	7	90	4
Other	396	2.41	58	3.31	14.65	53	5	325	13
Total	16443	100	1751	100	10.65	693	1058	9767	4925

^a^ **Note**: Ratio = (# of DE genes)/(# of All expressed genes). It represents what percentage of genes is differentially expressed. For a gene in each category, it is either a DE or Non_DE (not differential expression) gene, and then it is further broken into two classes based upon whether it overlaps with one or more genes transcribed from opposite strands. Therefore, the sum of the last four columns is equal to the total number of genes in that category

Next, we explored the association between differential analysis results and sequencing protocol. Every gene (dot) in Fig. 7 is either a DE (colored in red) or Non_DE (non-differential expression, colored in black) gene, and these genes are then further classified into one of two classes (i.e., “No” and “Yes”) based upon whether it overlaps with one or more genes transcribed from opposite strands. The overlap for each gene type is summarized in the last four columns in Table 1. The proportion of gene overlaps for all genes, DE genes, and Non_DE genes are shown in Fig. 8b. For protein coding, antisense and lincRNA gene types, the overlap is significantly higher in DE genes than in Non_DE genes. For instance, 87 % of antisense DE genes are overlapping genes, while only 60 % of antisense genes are overlapping genes in the Non_DE genes. For pseudogenes, no apparent association is observed, and confirmed by statistical test. To accept or reject the null hypothesis that differential expression and gene overlap are independent, the chi-square test was performed for the top four gene categories in Table 1. A contingency table was first prepared from the counts in the last four columns in Table 1, and then the *chisq.test* R function was called to evaluate the significance of the test. All tests report a P value lower than 2.2E-16, except for pseudogene (P value = 0.96).

As observed in Fig. 8, antisense genes are enriched substantially in differential expression, and this differential expression is strongly associated with gene overlap. The overwhelming majority of antisense DE genes show higher expression in stranded RNA-seq, and their expressions in non-stranded RNA-seq are quite often zero or very low. Antisense transcripts can act as regulatory elements in the regulation of gene expression [12], and a number of antisense transcripts are related to various human disorders [26]. A proper elucidation of the antisense transcriptome and its quantification will reveal their novel function in regulation of gene expression. Based on these observations, we have shown that the stranded RNA-seq is more effective than non-stranded RNA-seq in properly quantifying expression for antisense genes.

The ENCODE project recently performed a survey of publicly available expression data to identify transcribed pseudogenes and found over 800 pseudogenes with strong evidence of transcription [27].

Recent studies have shown that some pseudogenes are transcribed and contribute to cancer when dysregulated [28]. In particular, pseudogene transcripts can function as competing endogenous RNAs [29]. However, reliable quantification of pseudogene expression remains a challenging problem for a number of reasons. First, because parent genes and pseudogenes are highly similar in nucleotide sequence, short RNA-seq reads derived from one may align equally well to others. Such reads are fundamentally ambiguous in terms of their origin. Second, some reads may have nearly identical alignment to locations in the gene and pseudogene, and their mapping is often determined by the location with the least error in alignment. This strategy is unreliable and can result in an incorrect assignment of the read [29]. The enrichment of pseudogenes in differential analysis in Fig. 8a is hard to explain because the gene overlap from the opposite strand seems to not be the cause (see Fig. 8b). Of those 365 DE pseudogenes, 90 genes have higher expression in non-stranded RNA-seq, while 275 have higher expression in stranded RNA-seq. Usually the expression level for pseudogenes is not high. For those DE pseudogenes, the average expression is 3.9 RPKM across all eight samples, while for protein coding genes, the average is as high as 31.6 RPKM. We speculated the enrichment for pseudogenes might arise from (1) the read mapping uncertainty in pseudogenes, (2) the lower expression levels for pseudogenes, and (3) the additional bias introduced by sequencing protocols. We checked the read mapping profiles for some pseudogenes (unpublished results), and found that quite often those reads that mapped to pseudogenes have mismatches. Because of the intrinsic uncertainty in read mapping, we should be cautious about the gene quantification and differential analysis results for pseudogenes.

### Exemplary differential expression genes

**Fig. 9:**
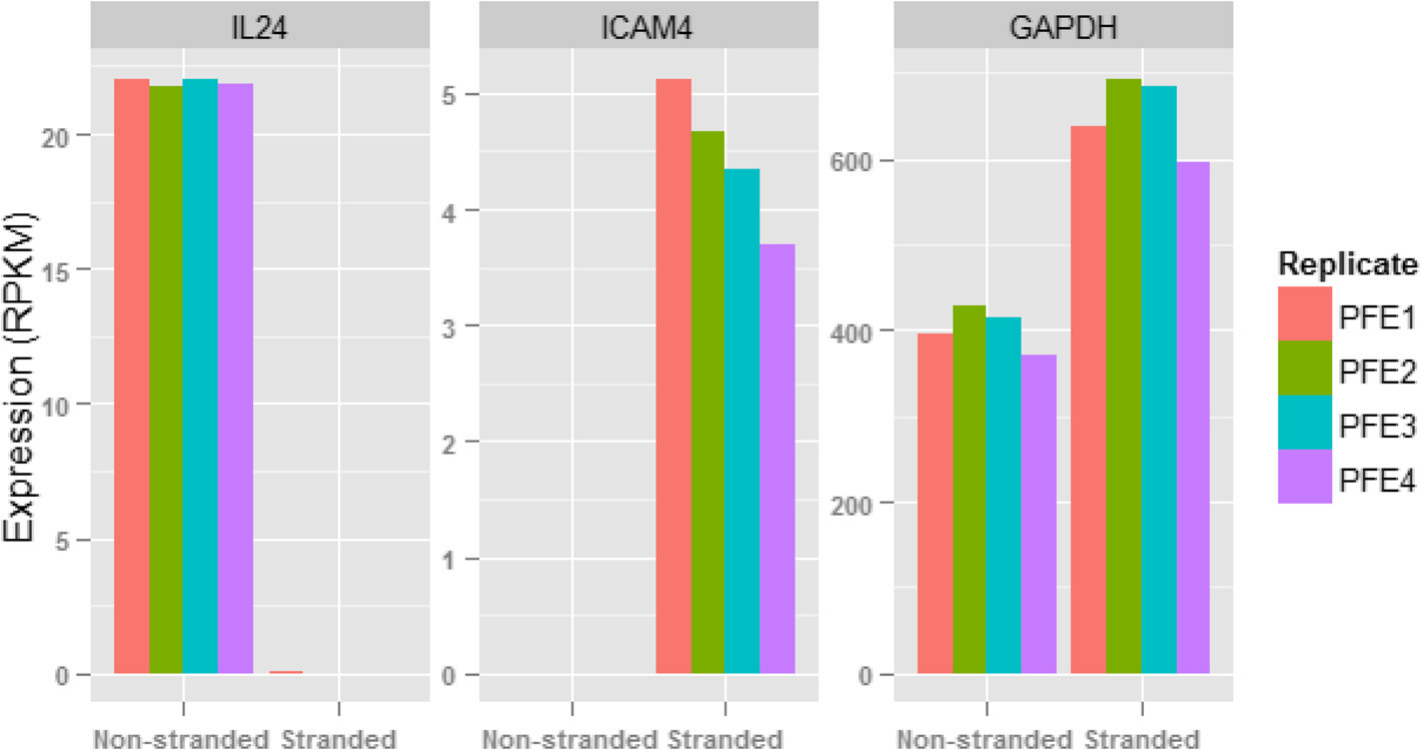
The gene expression of *IL24*, *ICAM4*, and *GAPDH* in stranded and non-stranded RNA-seq

For a given gene, if stranded and non-stranded RNA-seq report different expression levels, which one is more reliable? In principle, the stranded RNA-seq should be more accurate because additional information (i.e., the read direction) is used in gene quantification, and the ambiguous reads in overlapping genes transcribed from opposite strands are resolved and counted. Below, we selected a few example genes (i.e., *IL24*, *ICAM4*, and *GAPDH*) to demonstrate this point. The expressions for these three genes are shown in bar charts in Fig. 9.

**Fig. 10:**
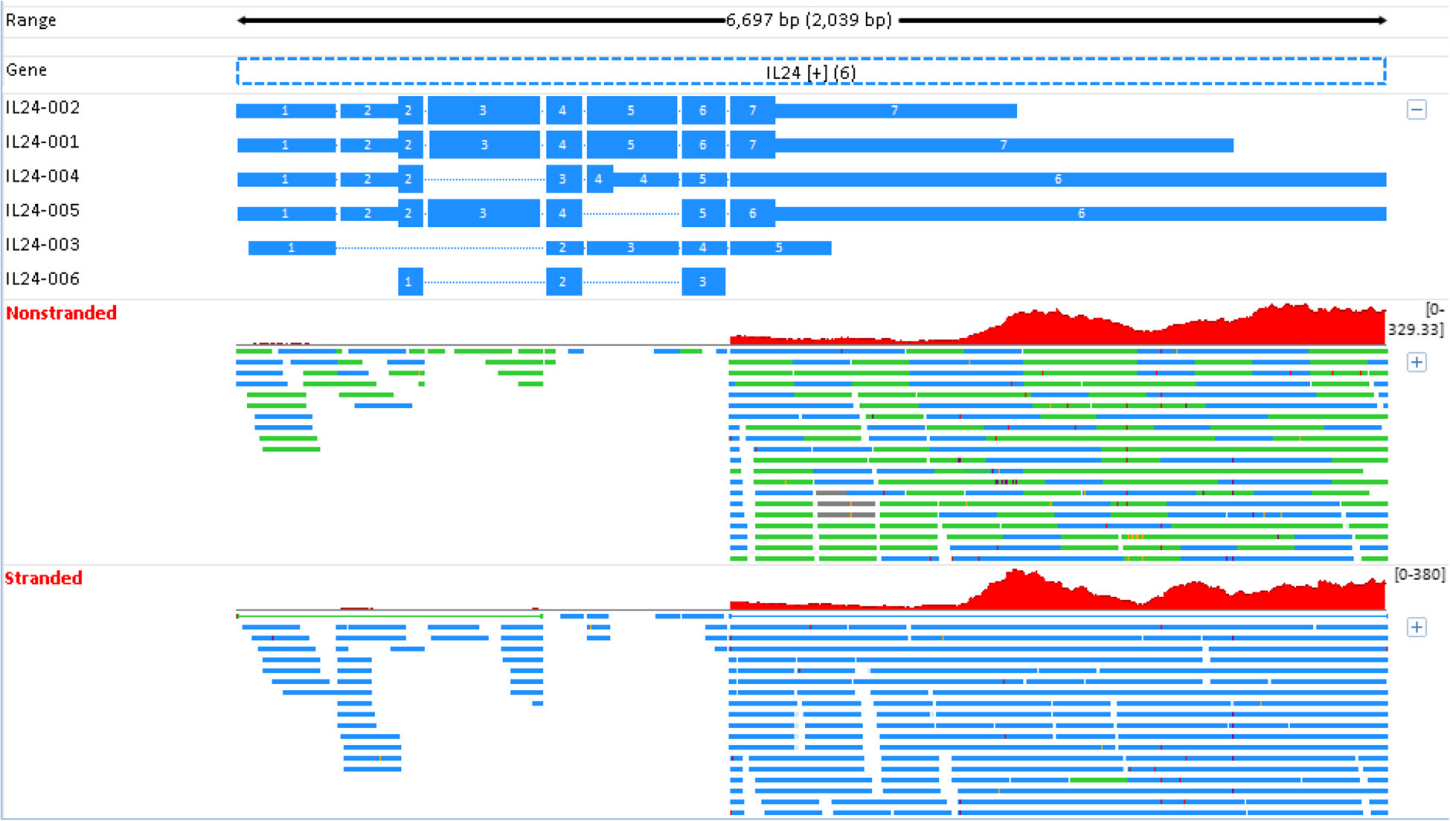
The mapping profiles for *IL24* in Replicate PFE1. In non-stranded RNA-seq, all reads mapped to *IL24* are counted regardless if they are in the forward or reverse strands. However, in stranded RNA-seq, nearly all reads are mapped to the “+” strand and thus not counted because these reads are not reverse complementary to IL24 in the “+” strand. However, the coverage pattern of sequence reads does not support the sequence reads mapped to the *IL24* genomic region that truly originate from this gene. All genes, transcripts, and sequence reads are colored in blue if they are in the “+” strand and colored in green if in the “−“ strand

Interleukin (IL) 24 is a secreted protein of the *IL10* family, and its expression has been identified in certain cell types. *In vivo*, *IL24* is predominantly expressed by skin tissue cells during inflammatory conditions, such as psoriasis [30]. In non-stranded RNA-seq, this gene has an expression level as high as approximately 22 RPKM in whole blood, but stranded RNA-seq reports no expression at all. The read mapping results in PFE1 are shown in Fig. 10. All genes, transcripts, and sequence reads in Fig. 10 are colored in blue if they are in the “+” strand, and colored in green if in the “−“ strand. Because too many reads were mapped to the *IL24* genomic region, particularly at the 3′UTR end, only a portion of mapped reads are shown in the plot. In non-stranded RNA-seq, all reads mapped to *IL24* are counted, regardless of their originating genomic strand. *IL24* is on the “+” strand, and thus a sequence read truly originating from *IL24* must be reverse complementary and mapped to the “−“ strand. Therefore, in stranded RNA-seq, only those reads mapped to the “−“ strand are counted. As can be seen, nearly all reads in stranded RNA-seq are mapped to the “+” strand (Fig. 10). As a result, those reads are not counted, clarifying why stranded RNA-seq reports no expression for *IL24*. The coverage pattern of sequence reads in Fig. 10 also does not support that they would originate from *IL24* either in stranded or non-stranded RNA-seq. The uniformity bias in RNA-seq does not explain the extremely uneven coverage pattern observed in Fig. 10. Moreover, this cytokine is not expected to have a high expression in whole blood RNA-seq [30]. The quantification of *IL24* expression in non-stranded RNA-seq is thus misleading. In contrast, the result in stranded RNA-seq is more reliable, and biologically makes sense with previous observations.

Because those reads in Fig. 10 are not derived from *IL24*, an obvious question is why so many reads are mapped to the genomic region of *IL24*. As we know, our current gene annotation is neither complete nor comprehensive, and it is likely that such reads originate from a novel gene at the opposite strand of *IL24*. We currently do not have a good explanation for these mapped reads. However, the scenario in Fig. 10 has shown that stranded RNA-seq is likely more powerful than non-stranded RNA-seq in detecting potentially novel unannotated transcripts from regions in which there is not a currently annotated gene.

**Fig. 11:**
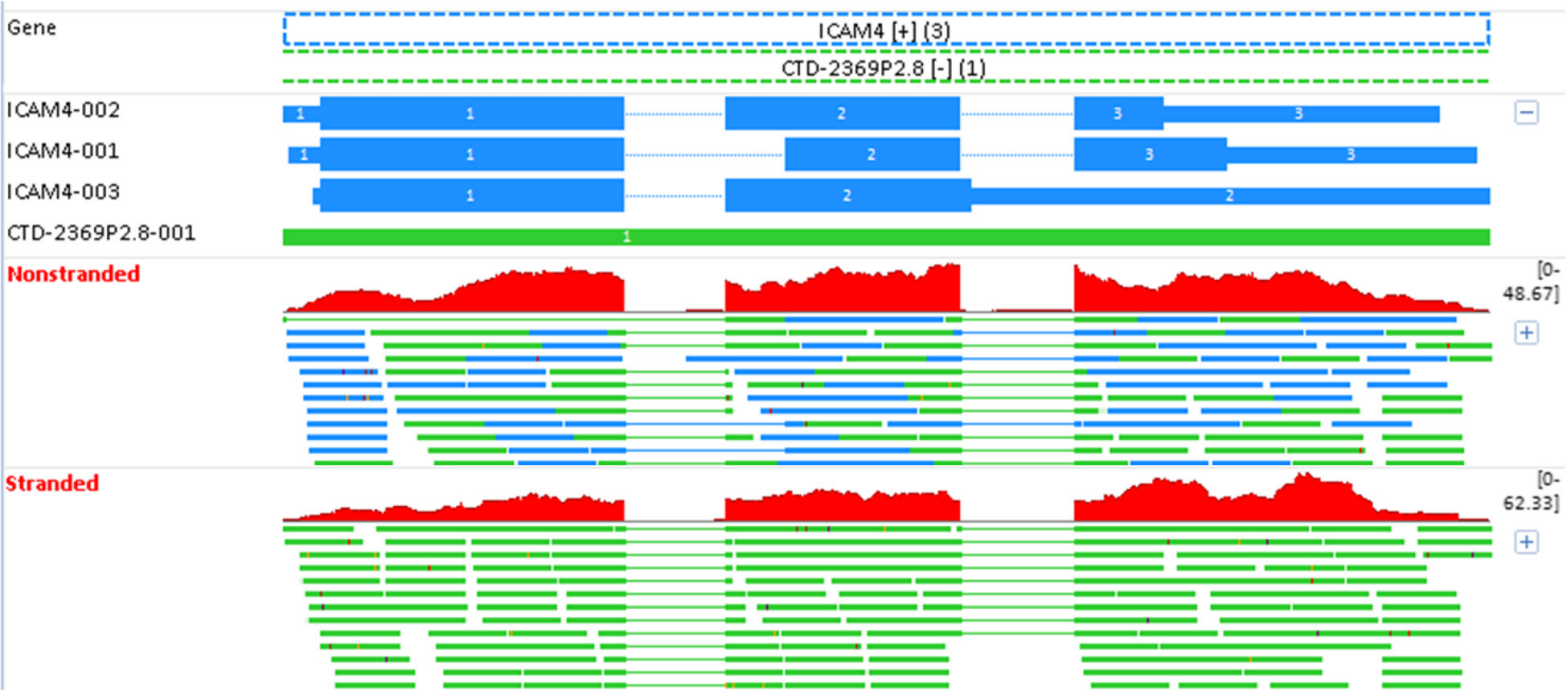
The mapping profiles for *ICAM4* (intercellular adhesion molecule 4) in Replicate PFE1. The gene *ICAM4* is on the “+” strand, and 100 % contained within *CTD-2369P2.8* in the “−“ strand. In non-stranded RNA-seq, the ambiguous reads in overlapping regions are excluded from counting, which explains why there is no expression for *ICAM4*. However, the ambiguous reads can be perfectly resolved in stranded RNA-seq. By considering the read direction, all reads can be counted to *ICAM4* because they are reverse complementary to *ICAM4*, but not *CTD-2369P2.8*

*ICAM4* (intercellular adhesion molecule 4) shows moderate expression in whole blood [31]. However, non-stranded RNA-seq reports no expression for this gene, and the reason is revealed in Fig. 11. *ICAM4* is encoded on the “+” strand, and it has three alternative splicing transcripts. It overlaps with another gene *CTD-2369P2.8* in the “−“ strand. *CTD-2369P2.8* is a manually annotated gene from the Havana (the Human and Vertebrate Analysis and Annotation) project, and it is longer than *ICAM4*. As observed in Fig. 11, *ICAM4* is 100 % contained within CTD-2369P2.8. In non-stranded RNA-seq, a read mapped to *ICAM4* is simultaneously aligned to *CTD-2369P2.8* as well. The ambiguous reads in overlapping regions are thus excluded from counting in FeatureCounts, and this explains the lack of expression for *ICAM4* on non-stranded RNA-seq. The ambiguous reads in overlapping genes in Fig. 11 can be perfectly resolved using stranded RNA-seq. By considering the read direction, all reads are assigned to *ICAM4* (but not *CTD-2369P2.8*), because they are all reverse complementary to *ICAM4*. According to our sequencing protocol, it is impossible for such reads to originate from *CTD-2369P2.8*. The gene expression in stranded RNA-seq also agrees with other supporting evidence [31], and is again more reliable than in non-stranded RNA-seq.

For the scenario in non-stranded RNA-seq in Fig. 11, it does not help if we use a different counting algorithm such as RSEM (RNA-Seq by Expectation-Maximization) [32]. Despite the fact that RSEM is capable of fully handling reads that map ambiguously or fall into the gene overlapping regions, it proportionally distributes ambiguous reads according to the number of unique reads in overlapping genes. If a gene is completely contained within another gene, it has no unique read at all. As a consequence, zero reads are counted to that gene. According to the theoretical calculation above, there are a total of 582 genes completely contained with other genes from opposite strands. In short, the read ambiguity in non-stranded RNA-seq in Fig. 11 cannot be resolved by a purely computational approach, and stranded RNA-seq is required in this scenario to determine correct gene expression.

*GAPDH* (glyceraldehyde-3-phosphate dehydrogenase) is a well-known housekeeping gene with very high expression in most cell types and tissues. Compared with stranded RNA-seq, its expression in non-stranded RNA-seq is in fact underestimated (Fig. 9). The reason for this underestimation can be easily understood when considering the gene overlap shown in Additional file 1: Figure S3. All of the ambiguous reads in the overlapping region originate only from *GAPDH* in stranded RNA-seq, thus the expression for *GAPDH* in stranded RNA-seq is more accurate than non-stranded.

## Conclusions

In this paper, we performed a side-by-side comparison of stranded and non-stranded RNA-seq, and investigated the gene overlap both in our practical whole blood RNA-seq dataset and from the theoretical perspective. Our study demonstrates that stranded RNA-seq provides a more accurate estimate of transcript expression compared with non-stranded RNA-seq and is therefore the recommended RNA-seq approach for all future mRNA-seq studies.

## Methods

We used various freely available open source tools and implemented an in-house pipeline for stranded and non-stranded RNA-seq data analyses (Fig. 2). The details on each step in the data generation and analyses are described below.

### Blood sample collection, RNA extraction, and globin mRNA depletion

Peripheral venous blood samples from five healthy male volunteers were collected in PAXgene Blood RNA tubes (PreAnalytiX GmbH, BD Biosciences, Mississauga, ON, Canada). Blood was pooled across subjects to create a single pooled sample. This pooled blood was dispensed into a set of approximately 10-mL aliquots. Total RNA was extracted from four aliquots of pooled blood using the PAXgene Blood RNA Kit (cat# 762164, Qiagen, Chatsworth, CA, USA) according to the manufacturer's protocol. The yield and quality of the isolated RNA were assessed using a NanoDrop8000 Spectrophotometer (Thermo Scientific, Wilmington, DE, USA) and Agilent 2100 Bioanalyzer (Agilent Technologies, Santa Clara, CA, USA), respectively. An aliquot of 1.5 mg of each RNA was further processed with a GlobinClear kit (cat# AM1980, Life Technologies, Carlsbad, CA, USA) to remove globin mRNA. After globin mRNA depletion, the quality and yield of the RNA were assessed again using an Agilent 2100 Bioanalyzer. Six hundred nanograms of RNA (post-GlobinClear) were divided into two 300 ng aliquots, with one aliquot submitted to stranded RNA-seq processing and the second aliquot submitted to non-stranded RNA-seq processing.

### cDNA library construction and sequencing

For stranded RNA-seq, cDNA libraries were prepared with a TruSeq stranded mRNA library prep Kit (cat# RS-122-2101, Illumina, San Diego, CA , USA). For non-stranded RNA-seq, cDNA libraries were prepared with a TruSeq RNA sample preparation kit v2 (cat# RS-122-2001, Illumina). The resulting eight libraries were sequenced on a HiSeq 2000 (Illumina) using a paired-end run (2 × 100 bases). A minimum of 60 M reads were generated from each library. The clean raw sequence reads in FASTQ format were analyzed using the pipeline in Fig. 2.

### Mapping and counting

The human genome database and gene annotation database were used to map and count sequence reads. Gencode Release 19 was downloaded from http://www.gencodegenes.org/releases/19.html. The reads were mapped to the hg19 reference genome using STAR v2.4.0h [15]. The detail parameters for the STAR run were “*--runThreadN 8 --alignSJDBoverhangMin 1 --outReadsUnmapped Fastx --outFilterMismatchNoverLmax 0.05 --outFilterScoreMinOverLread 0.90 --outFilterMatchNminOverLread 0.90 --alignIntronMax 1000000 --outSAMtype BAM SortedByCoordinate*”. The mapping was performed on the Pfizer High Performance Computing cluster. The mapping summaries, such as the percentage of reads that were uniquely mapped, multiple mapped, or unmapped, were then collected from the log files of STAR runs (see Results).

To count reads mapped to individual genes in Gencode, the program featureCounts [16] was used. FeatureCounts assigns a read to a feature (a gene) or labels it as matching to no feature or as ambiguous if it matches more than one feature and it cannot determine which one it is. The parameters in featureCounts run were “*-p -T 4 -F GTF -a hg19.gencode.v19.gtf -t exon -g gene_id -s****$Strand****-B -C --minReadOverlap 60*” (note **$Strand** was set to 0 for non-stranded RNA-seq, and 2 for dUTP second strand marking RNA sequencing protocol). Only uniquely mapped reads are used in the counting step. Like the mapping step above, the counting metrics were collected from the summary file of each featureCounts run. Genes that have expression levels less than 1 CPM were labeled as low expressed. If a gene had zero or low expression across all eight samples, it was omitted from the correlation and differential expression analysis. This filtering step was included to reduce the false positives in the differential analysis [33].

### Differential expression analysis

A counts table was generated by featureCounts and then used for the DE analysis. The differential analysis was performed by R packages edgeR 3.8.5 [17] and Limma/voom 3.22.4 [18]. We compared the stranded versus non-stranded sequencing groups. All genes with a fold change greater than 1.5 and a Benjamini-Hochberg adjusted p-value smaller than 0.05 were reported as DE genes.

### Theoretical estimation of gene overlapping at the same and opposite strands

The estimation was performed by R package GenomicFeatures 1.18.3 [34]. First, a transcript database (TxDB) was created from the Gencode annotation in GTF format by calling R function *makeTranscriptDbFromGFF*. We then extracted all exons from TxDb and grouped them by gene. According to strand information, the genes in each chromosome were divided into two groups. The overlaps at the same and opposite strands were quantified at both gene and nucleotide base levels (see Fig. 4). For each pair of overlapping genes, for example G1 and G2, the lengths for flattened exons were calculated and the short gene was selected for calculating the ratio of overlapping. The histogram and cumulative distribution of overlap were quantified (Fig. 5).

## Consent

The protocol for the Pfizer Research Support Program to collect blood samples from volunteer donors was approved by the Schulman Associates Institutional Review Board (IRB#201065670; http://www.sairb.com/Pages/home.aspx). Written informed consent was obtained from all volunteer blood donors for the research described and potential publication thereof. A copy of the written consent is available for review by the Editor of this journal. Samples from individuals were coded at the time of collection and then pooled prior to data generation, removing any possible association of analytical measurements with a single donor.

## Availability of supporting data and script

All the raw sequencing reads have been submitted to the NCBI Sequence Read Archive and are available under accession SRP056985.

The R script to estimate the gene overlap is attached as Additional file 1: Script 1.

## Additional file

Additional file 1:Table S1. Reports the related metrics for all eight RNA-seq samples, including library sizes, the mapping summaries, and the counting summaries. **Tables S2.** and **S3.** Tabulate the overlapping summaries of Gencode V19 annotation database at both the gene and the nucleotide base levels, respectively. **Figures S1.** and **S2.** Show all-against-all scatter plots of gene expression profile among RNA-seq samples sequenced by stranded and non-stranded protocols, respectively. **Figure S3.** Explains why the expression level for *GAPDH* (a well-known housekeeping gene) is underestimated in non-stranded RNA-seq. **Script 1.** Contains the R script to estimate the gene overlap in Gencode Release 19. (PDF 429 kb)
