# Supplementary material for: Comparison of stranded and non-stranded RNA-seq transcriptome profiling and investigation of gene overlap
Source: BMC Genomics. 2015 Sep 3;16(1):675. doi: 10.1186/s12864-015-1876-7 (PMC4559181; doi:10.1186/s12864-015-1876-7)
Supplement: Additional file 1:Table S1. — Reports the related metrics for all eight RNA-seq samples, including library sizes, the mapping summaries, and the counting summaries. Tables S2. and S3. Tabulate the overlapping summaries of Gencode V19 annotation database at both the gene and the nucleotide base levels, respectively. Figures S1. and S2. Show all-against-all scatter plots of gene expression profile among RNA-seq samples sequenced by stranded and non-stranded protocols, respectively. Figure S3. Explains why the expression level for GAPDH (a well-known housekeeping gene) is underestimated in non-stranded RNA-seq. Script 1. Contains the R script to estimate the gene overlap in Gencode Release 19. (PDF 429 kb) [file 12864_2015_1876_MOESM1_ESM.pdf]

Supplementary Table 1. The summary of sequence reads mapping and counting

Sample	Mapping				Counting			
	#PE Reads	Unique (%)	multiple (%)	unmapped (%)	#Reads	Gene (%)	Ambiguity (%)	No_Feature (%)
PFE1_S	62893222	88.44	3.58	7.98	55621208	88.71	2.94	8.35
PFE2_S	61184208	87.67	3.49	8.84	53637345	88.24	2.96	8.80
PFE3_S	62038783	89.27	3.51	7.22	55380840	88.95	2.94	8.11
PFE4_S	68270467	89.82	3.48	6.70	61320461	88.50	2.88	8.61
PFE1_NS	61617213	90.06	3.47	6.47	55490179	86.79	6.17	7.04
PFE2_NS	63096109	90.28	3.35	6.37	56961512	86.70	6.12	7.18
PFE3_NS	61433361	89.30	3.31	7.39	54862845	86.78	6.06	7.16
PFE4_NS	63979506	91.12	3.36	5.52	58299380	86.38	5.99	7.63

Supplementary Table 2. Summary of gene overlaps in Gencode Release 19 at the gene level.
(**Note**: Columns 3-5: the number overlapping genes in **SS** (Same Strand), **OS** (Opposite Strand), and **AS** (Any Strand); and Columns 6-8 are the corresponding percentages)

Chr	Gene	Gene_SS_Overlap	Gene_OS_Overlap	Gene_AS_Overlap	Gene_SS_Pct	Gene_OS_Pct	Gene_AS_Pct
chr1	5363	464	995	1322	8.65	18.55	24.65
chr2	4047	325	770	1011	8.03	19.03	24.98
chr3	3101	270	627	806	8.71	20.22	25.99
chr4	2563	134	339	455	5.23	13.23	17.75
chr5	2859	198	517	670	6.93	18.08	23.43
chr6	2905	218	532	686	7.5	18.31	23.61
chr7	2876	268	536	709	9.32	18.64	24.65
chr8	2386	129	408	516	5.41	17.1	21.63
chr9	2323	170	336	471	7.32	14.46	20.28
chr10	2260	181	409	535	8.01	18.1	23.67
chr11	3208	313	671	891	9.76	20.92	27.77
chr12	2818	254	651	829	9.01	23.1	29.42
chr13	1217	71	135	195	5.83	11.09	16.02
chr14	2244	205	449	577	9.14	20.01	25.71
chr15	2080	243	487	652	11.68	23.41	31.35
chr16	2343	330	692	900	14.08	29.53	38.41
chr17	2903	389	859	1098	13.4	29.59	37.82
chr18	1127	61	213	256	5.41	18.9	22.72
chr19	2910	523	799	1185	17.97	27.46	40.72
chr20	1317	114	211	297	8.66	16.02	22.55
chr21	736	55	104	144	7.47	14.13	19.57
chr22	1263	180	277	409	14.25	21.93	32.38
chrX	2392	149	189	323	6.23	7.9	13.5
chrY	542	58	31	82	10.7	5.72	15.13
total	57783	5302	11237	15019	9.18	19.45	25.99

Supplementary Table 3. Summary of gene overlaps at nucleotide base level in Gencode Release 19. (**Note**: Columns 3-5: the number overlapping nucleotide bases in **SS** (Same Strand), **OS** (Opposite Strand), and **AS** (Any Strand); and Columns 6-8 are the corresponding percentages)

Chr	Base	NT_SS_Overlap	NT_OS_Overlap	NT_AS_Overlap	NT_SS_Pct	NT_OS_Pct	NT_AS_Pct
chr1	12109200	291385	415134	703246	2.41	3.43	5.81
chr2	9324128	219469	338690	556475	2.35	3.63	5.97
chr3	7385592	190215	248804	433196	2.58	3.37	5.87
chr4	5404937	69998	137310	206982	1.3	2.54	3.83
chr5	6256617	160314	174236	337590	2.56	2.78	5.4
chr6	6171281	164353	199584	361907	2.66	3.23	5.86
chr7	6223179	190168	203084	386694	3.06	3.26	6.21
chr8	4858915	92579	158756	249501	1.91	3.27	5.13
chr9	4931735	145989	129622	272714	2.96	2.63	5.53
chr10	4882040	141487	167692	306491	2.9	3.43	6.28
chr11	7115007	184539	274828	454535	2.59	3.86	6.39
chr12	6599073	164301	274696	432895	2.49	4.16	6.56
chr13	2281816	60604	41966	102225	2.66	1.84	4.48
chr14	4246521	172817	168422	335954	4.07	3.97	7.91
chr15	4697387	166647	204126	365736	3.55	4.35	7.79
chr16	5515892	284033	342292	619321	5.15	6.21	11.23
chr17	7053533	360672	367098	720967	5.11	5.2	10.22
chr18	2359228	27410	84938	111862	1.16	3.6	4.74
chr19	6864790	381607	321724	696352	5.56	4.69	10.14
chr20	2697355	78513	65904	143902	2.91	2.44	5.33
chr21	1497852	47285	40216	86899	3.16	2.68	5.8
chr22	2876015	139543	118006	254257	4.85	4.1	8.84
chrX	4306404	69695	66604	135545	1.62	1.55	3.15
chrY	646073	18955	3718	22572	2.93	0.58	3.49
total	126304570	3822578	4547450	8297818	3.03	3.6	6.57

Supplementary Figure 1. All-against-all scatter plots among all 4 **stranded** RNA-seq samples (Note: the x- and y-axis represent log2(RPKM))

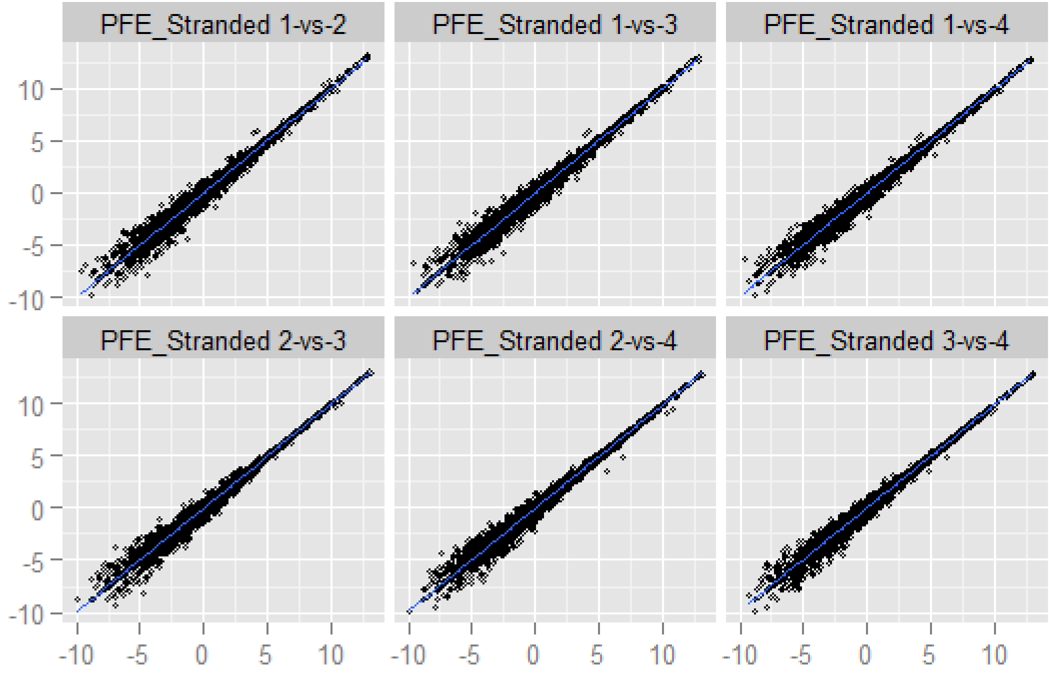

Supplementary Figure 2. All-against-all scatter plots among all 4 **non-stranded** RNA-seq samples (Note: the x- and y-axis represent log2(RPKM))

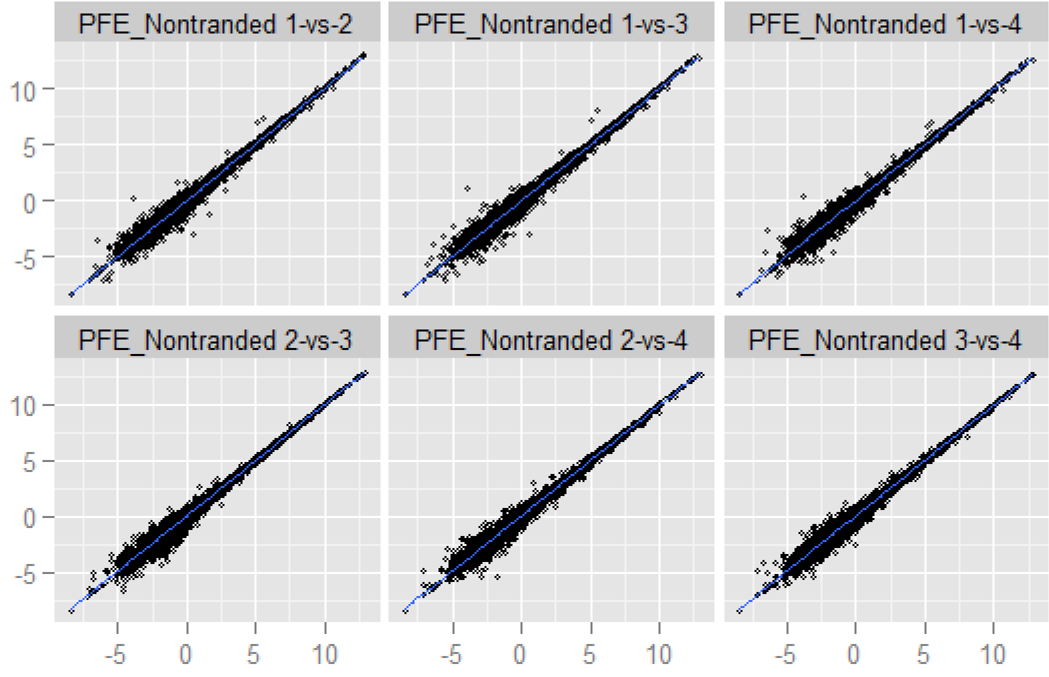

Supplementary Figure 3. The breakdown of all genes in Gencode Release 19 and the breakdown of differential expression (DE) genes. Antisense and pseudogene are enriched in DE genes.

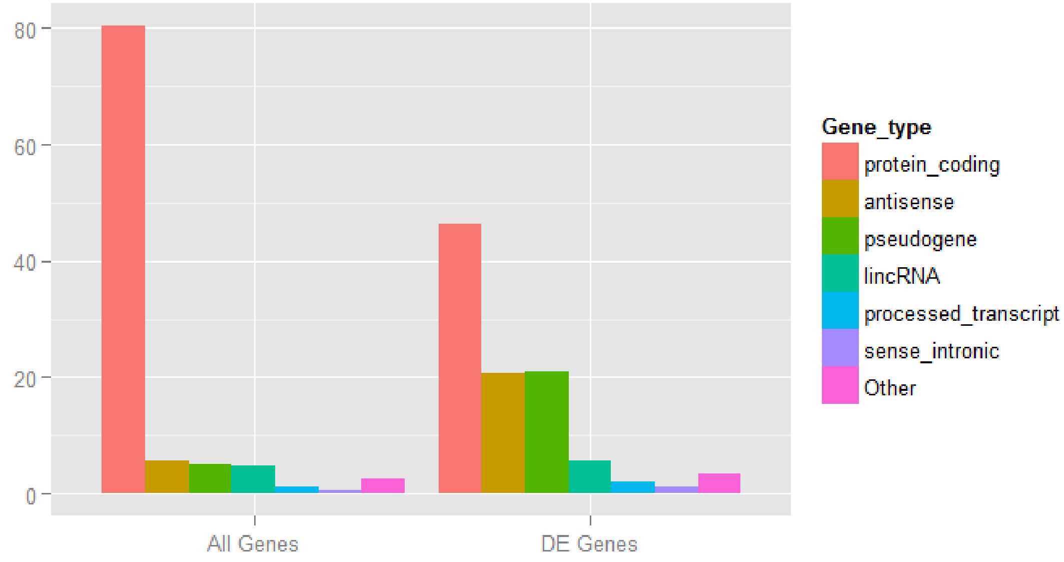

Supplementary Figure 4. The expression level for GAPDH, a well-known housing keeping gene, is underestimated by ~50% in nonstranded RNA-seq. In stranded RNA-seq, all those reads mapped to the overlapping regions turn out to truly originate from GAPDH, and thus a more accurate quantification is obtained from stranded RNA-seq.

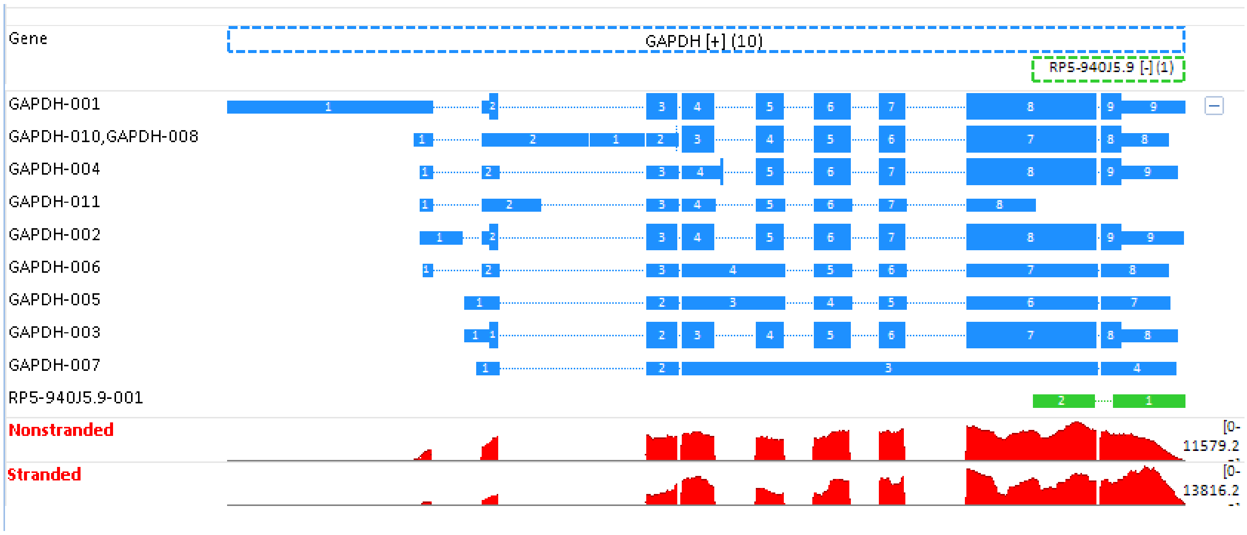

Supplementary Script 1. R code for gene overlap

```
#############################################################################
#
# Theoretical estimation of gene overlap for Gencode Release 19
#
# Author:  Shanrong Zhao
# Release date:   April 1, 2015
#
#############################################################################

## ----loadGenomicFeatures----------------------------------------------------
--------------
library("GenomicFeatures")

setwd("C:\\Pfizer_Project\\Stranded-Specific\\Manuscript\\data\\Gencode")

## ----loadDb---------------------------------------------------------------
# download Gencode V19 from http://www.gencodegenes.org/releases/19.html
# upzip and save the file as "hg19.gencode.v19.gtf"
txdb <- makeTranscriptDbFromGFF(file="hg19.gencode.v19.gtf", format="gtf")
txdb

#saveDb(txdb, file="gencode.v19.sqllite")
#gencode.v19 <- "gencode.v19.sqllite"
#txdb <- loadDb(gencode.v19)


## ----seqlevels-------------------------------------------------------------
chrs <- paste("chr", c(1:22,"X","Y"), sep="")
gene.overlap.details <- list();
gene.overlap.summaries <- list()
gene.overlap.pairs <- list()  #detail for every pairs of overlapping genes

# process the genes chromosome by chromosome
for (chr in chrs) {
      txdb <- restoreSeqlevels(txdb)
      seqlevels(txdb, force=TRUE) <- chr

      # exons by genes, and split by strand
      exons <- reduce(exonsBy(txdb, by = "gene"))

      exons.p <- exons[strand(exons) == "+",]
      exons.p <- exons.p [elementLengths(exons.p) > 0,]

      exons.m <- exons[strand(exons)=="-",]
      exons.m <- exons.m [elementLengths(exons.m) > 0,]

      #gene genomic info
      exons <- c(exons.p, exons.m)
      strands <- c(rep("+", length(exons.p)), rep("-", length(exons.m)))
      genes <- names(exons)

      data <- as.data.frame(range(exons))
      data$exon_no <- elementLengths(exons)
      data$exon_length <- sum(width(exons))
```

```
#a better and more informative way
hits <- as.matrix(findOverlaps(exons, exons, ignore.strand = TRUE))
hits <- hits[hits[,1] != hits[,2],]
n = length(genes)
overlaps = data.frame(SS=rep(0,n),OS=rep(0,n), AS=rep(0,n),
      SS_genes=rep("",n), OS_genes=rep("",n),stringsAsFactors=FALSE)

for (i in 1:nrow(hits)) {
      g1 = hits[i,1]
      g2 = hits[i,2]
      if ( g1==g2 ) { next }        #remove self overlap with self

      gene <- genes[g2]

      if ( strands[g1] == strands[ g2] ) {
            overlaps$SS[g1] <- overlaps$SS[g1]+1

            if (overlaps$SS_genes[g1] == "") {
                  overlaps$SS_genes[g1] = gene
            } else {
                  overlaps$SS_genes[g1] =
                        paste(overlaps$SS_genes[g1],gene, sep=";")
            }
            overlap.tag <- "SS"

      } else {
            overlaps$OS[g1] <- overlaps$OS[g1]+1

            if (overlaps$OS_genes[g1] == "") {
                  overlaps$OS_genes[g1] = gene
            } else {
                  overlaps$OS_genes[g1] =
                        paste(overlaps$OS_genes[g1],gene, sep=";")
            }
            overlap.tag <- "OS"
      }

      #overlap pair details
      pair1 <- paste(genes[g1],genes[g2], sep=":")
      pair2 <- paste(genes[g2],genes[g1], sep=":")

      #note: pair is recorded only once
      if (is.null(gene.overlap.pairs[[pair1]]) &&
                  is.null(gene.overlap.pairs[[pair2]])) {
            ex1 <- exons[[ genes[g1] ]]
            ex2 <- exons[[ genes[g2] ]]
            ex12 <- intersect(ex1,ex2,ignore.strand = TRUE)

            l1 <- sum(width(ex1))
            l2 <- sum(width(ex2))
            l12 <- sum(width(ex12))
            gene.overlap.pairs[[pair1]] <-
               data.frame(g1=genes[g1],g2=genes[g2],strand=overlap.tag,
                  len1=l1, len2=l2, overlap=l12)
      }
```

```
        }

        overlaps$AS <- overlaps$OS + overlaps$SS
        colnames(overlaps)[1:3] <- c("SS_overlap","OS_overlap","AS_overlap")
        data <- cbind(data,overlaps)
        gene.overlap.details[[chr]] <- data

        gene.total <- length(exons.p) + length(exons.m)
        gene.overlap <- apply( overlaps[,1:3]>0, 2, sum)

        nt.total = sum(data$exon_length)

        nt.overlap.pp <- table(coverage(exons.p))[-c(1,2)]
        nt.pp <- sum(nt.overlap.pp * c(1:length(nt.overlap.pp)+1) )

        nt.overlap.mm <- table(coverage(exons.m))[-c(1,2)]
        nt.mm <- sum(nt.overlap.mm * c(1:length(nt.overlap.mm)+1) )

        nt.overlap <- table(coverage ( exons))[-c(1,2)]
        nt.both <- sum( nt.overlap * c(1:length(nt.overlap)+1) )

        nt.strand = nt.pp + nt.mm

        # it is trick to calculate the overlaps from opposite strands.
        # Below we flatten the exons in the same strand, and then calculate
        # the overlap
        exons <- reduce(exons(txdb))
        nt.overlap.opposite <- table(coverage ( exons))[-c(1,2)]
        nt.opposite <- sum( nt.overlap.opposite * 2 )

        nt.overlap <- c(nt.strand,nt.opposite,nt.both)

        gene.overlap.summaries[[chr]] <- c(gene.total, gene.overlap,
              gene.overlap*100/gene.total,
              nt.total, nt.overlap , nt.overlap*100/nt.total)
}


#
#write out overlap detail
#
overlap.details <- do.call(rbind,gene.overlap.details)
colnames(overlap.details)[1:3] <- c("group","gene", "chr")
overlap.details = cbind(gene=overlap.details$gene, overlap.details[,-c(1,2)])

overlap.details <- overlap.details[ order(overlap.details$gene),]
write.csv(overlap.details, file="overlap.details.csv", row.names=F, quote=F)


#
#overlap pair details
#
overlap.pairs <- do.call(rbind,gene.overlap.pairs)
overlap.pairs$pct1 <- overlap.pairs$overlap/overlap.pairs$len1
overlap.pairs$pct2 <- overlap.pairs$overlap/overlap.pairs$len2
overlap.pairs$overlap_pct <- pmax(overlap.pairs$pct1,overlap.pairs$pct2)
```

```
overlap.pairs[,c("pct1","pct2","overlap_pct")] <-
      round(overlap.pairs[,c("pct1","pct2","overlap_pct")]*100, digits=2)
write.csv(overlap.pairs, file="overlap.pairs.csv", row.names = F, quote = F)

overlap.os <- overlap.pairs[ overlap.pairs$strand=="OS",]
overlap.ss <- overlap.pairs[ overlap.pairs$strand=="SS",]


#
# plot overlap among overlapping pair of genes
#
old.par <- par(mfrow=c(2, 2))
hist(overlap.ss$overlap_pct, xlab="Overlap (%)", las=1,
      main="a) Same strand")
hist(overlap.os$overlap_pct, xlab="Overlap (%)", las=1,
      main="b) Opposite strand")

plot(sort(overlap.ss$overlap_pct), (1:nrow(overlap.ss))/nrow(overlap.ss)*100,
            las=1,type = 's', ylim = c(0, 100), xlab="Overlap (%)",
            ylab="The cumulative distribution (%)",
            main="c) Same strand")
abline(h=20, lty=2)
abline(h=50, lty=2, col="red")
points(median(overlap.ss$overlap_pct), 50, col="red")
abline(h=80, lty=2)


plot(sort(overlap.os$overlap_pct),(1:nrow(overlap.os))/nrow(overlap.os)*100,
            las=1,type = 's', ylim = c(0, 100), xlab="Overlap (%)",
            ylab="The cumulative distribution (%)",
            main="d) Opposite strand")
abline(h=20, lty=2)
abline(h=50, lty=2, col="red")
points(median(overlap.os$overlap_pct), 50, col="red")
abline(h=80, lty=2)


#
# overlap summary
#
overlap.summary <- do.call(rbind,gene.overlap.summaries)
colnames(overlap.summary) <-
c("Gene","Gene_SS_Overlap","Gene_OS_Overlap","Gene_AS_Overlap",
      "Gene_SS_Pct","Gene_OS_Pct","Gene_AS_Pct",
      "Base","NT_SS_Overlap","NT_OS_Overlap","NT_AS_Overlap",
      "NT_SS_Pct","NT_OS_Pct","NT_AS_Pct")

All <- apply(overlap.summary, 2, sum)
All[5:7] <- All[2:4]*100/All[1]
All[12:14] <- All[9:11]*100/All[8]
overlap.summary <- rbind(overlap.summary,All)

overlap.summary[,5:7] <- round(overlap.summary[,5:7], digits=2)
overlap.summary[,12:14] <- round(overlap.summary[,12:14], digits=2)
write.csv(overlap.summary, file="overlap.summary.csv")
```

```
#
# plot overlap summary at gene and nucleotide base levels
#
library(ggplot2)
library(reshape2)

rownames(overlap.summary) <- sub("chr","", rownames(overlap.summary))
gene.summary <-cbind(data.frame(chr=rownames(overlap.summary)),
overlap.summary[,c(5,6)])
colnames(gene.summary) <- c("Chromosome","Same_Strand","Opposite_Strand")

nt.summary <-cbind(data.frame(chr=rownames(overlap.summary)),
overlap.summary[,c(12:13)])
colnames(nt.summary) <- c("Chromosome","Same_Strand","Opposite_Strand")

#convert wide to long form
plot.summary <- function (data, main_title) {
      data <- melt(data, id.vars=colnames(data)[1],
            measure.vars=colnames(data)[-1],
            variable.name="Overlap",
            value.name="Percent"
            )
      data$Chromosome <- factor(data$Chromosome,
                  levels=unique(data$Chromosome))

      g1<- ggplot(data,aes(x=Chromosome, y=Percent))+
            geom_bar(stat="identity",aes(fill=Overlap),
            position=position_dodge(0.8))+
            xlab("Chromosome") + ylab("Percentage of overlap") +
            theme(axis.text=element_text(size=8, face="bold"),
                  axis.title=element_text(size=9,face="bold")) +
                  ggtitle(main_title) +
            guides(fill = guide_legend(
                  title.theme = element_text(size=9,angle=0,,face="bold"),
                  label.theme = element_text(size=8,angle=0,face="bold"),
                              ))
      g1
}

gene.plot <- plot.summary( gene.summary, "The overlap at the gene level")
nt.plot <-plot.summary( nt.summary,"The overlap at the nucleotide base level")

gene.plot
nt.plot
```
